# Scalp Bogginess in the Presence of Lichen Planopilaris: Isolated Occurrence or Reactionary Process?

**DOI:** 10.7759/cureus.73699

**Published:** 2024-11-14

**Authors:** Victoria S Jiminez, Eleanor Workman, Omid Jalali

**Affiliations:** 1 Heersink School of Medicine, University of Alabama at Birmingham, Birmingham, USA; 2 Dermatology, Baylor College of Medicine, Houston, USA

**Keywords:** lichen planopilaris, lipedematous scalp, scalp edema, scarring alopecia, spongy scalp syndrome

## Abstract

Lipedematous scalp (LS) and lipedematous alopecia (LA) are rare conditions involving focal or diffuse hyperplasia within subcutaneous adipose tissue of the scalp. Little is known regarding the etiology of these conditions, and there is no consensus on management strategies. Overall, the condition is benign and often isolated. However, there have been reported cases of co-existing scalp edema in the presence of another pathogenic process involving the scalp and hair. The authors present a case of significant scalp edema in a middle-aged female who presented with lichen planopilaris (LPP) of the bifrontal scalp of four years duration. The patient had diffuse thinning of the frontal scalp with a perifollicular scale, and further examination revealed significant edema and bogginess of the remainder of the scalp. A punch biopsy of the frontal scalp was obtained and consistent with LPP. LA is typically seen in areas where both scalp thickening and hair loss are present and has little or no histologic inflammation. This case highlights the presence of scalp edema in a patient with inflammatory scarring hair loss, which has not been previously reported. This may suggest a new variant of this spectrum of hyperplastic or edematous conditions of the scalp as a reactive process in the presence of another primary, inciting condition such as scarring alopecia. Additionally, LA should be a diagnosis of exclusion after ruling out treatable alopecic conditions such as LPP.

## Introduction

Lipedematous scalp (LS) is a rare condition of unknown etiology characterized by subcutaneous adipose hyperplasia, which clinically results in the scalp's thickness and bogginess [1]. Lipedematous alopecia (LA) occurs when hair loss and scalp bogginess are present. Knowledge surrounding these disorders is primarily based on case reports, can occur at any age, and has been postulated to have hormonal and genetic components [1]. Those with LA typically have localized hair loss of the vertex and occipital scalp [1]. However, some cases progress from the initial site to involve the entire scalp [1]. We present the case of a patient with a four-year history of bifrontal scarring hair loss who was found to have diffuse bogginess of the scalp.

## Case presentation

A 47-year-old female with no significant past medical history presented for alopecia. The patient had an insidious onset of hair thinning of the frontal scalp for approximately four years, with progression in recent months. She had not been evaluated for this prior to her presentation and received no treatments. No subjective symptoms such as pruritis or pain were present, and the patient denied excessive manipulation of the hair or pulling.

**Figure 1:**
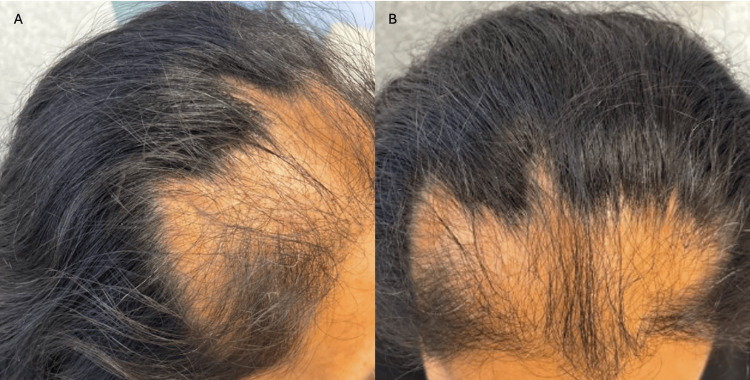
Vertex (A) and lateral (B) views of bifrontal alopecia

**Figure 2:**
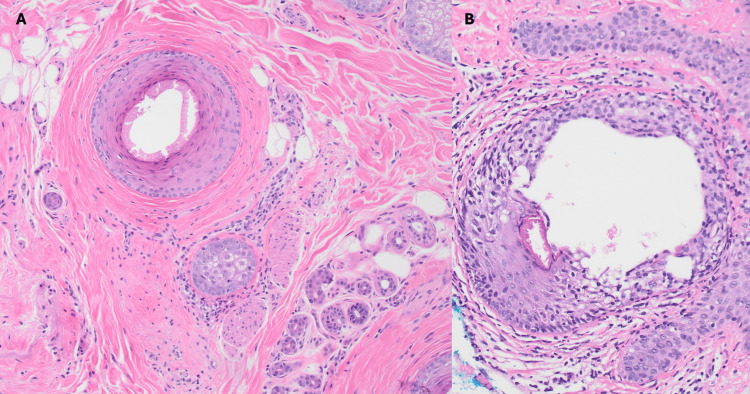
Horizontal (A) and vertical (B) histologic images of scalp biopsy with follicular scarring and inflammation (200x)

On examination, the patient had decreased hair density of the bifrontal scalp in a banded distribution with a perifollicular scale (Figure 1). On further examination, palpation of the scalp revealed diffuse boggy induration of the remainder of the scalp without associated hair abnormalities. The hair pull test was negative, and no erythema or scale was seen in the vertex, parietal, or occipital scalp. A punch biopsy of the frontal scalp was performed. It revealed focal lichenoid infiltration of lymphocytes predominantly in the infundibular region of the hair follicle with fibrosis, consistent with lichen planopilaris (LPP) (Figure 2). A lipid panel and hemoglobin A1C prior to presentation were within normal limits. The patient was started on hydroxychloroquine 200 mg daily and fluocinonide 0.05% solution.

## Discussion

LA and LS are benign conditions that follow a chronic, undulating course. It is estimated that most affected individuals are Black/African American or White females, and there have been no reports of this in Hispanic/Latino individuals [2,3]. Clinical, histopathological, and radiologic findings can support the diagnosis of LS and LA [1]. Other lipomatosis conditions have been reported and are defined as hyperplastic, not neoplastic, proliferations of mature fats in certain parts of the body [3]. The pathogenesis is hypothesized to involve hormonal regulatory pathways, lending support to the observed female preponderance [4]. Specifically, leptin has been implicated as a potential hormone that regulates fat mass and distribution [2,4]. Metabolic diseases have been postulated to be concomitant in patients with LS/LA and may lend insight into leptin dysregulation. Some cases of LS/LA have also suggested a possible relationship between autoimmune/autoinflammatory conditions. Other cases have reported patients with LS and discoid or systemic lupus erythematosus, alopecia areata, and scalp psoriasis [5-8].

The LS is characterized histologically by the thickening of the subcutaneous adipose tissue, and the diagnosis hinges on the lack of significant inflammatory infiltrate [1,4]. The patient presented here only had a biopsy of the area of alopecia that was concerning to her, and therefore, a definitive histologic diagnosis of LS cannot be confirmed. The presence of substantial clinical scalp edema with concomitant LPP of the frontal hairline may represent a new variant of the LS/LA spectrum. Cases of LA reported previously did not have clinical evidence of inflammation and histologically had normal follicular structures with sparse or no inflammatory infiltrates [6]. Areas of hair loss also typically coincide with the distribution of the scalp thickening, unlike this case [6]. Many of the existing reported cases of LS also had only focal and/or unilateral thickening of one or two scalp regions, such as the vertex, parietal, and occipital scalp [1,6,9,10]. Two have had a distribution similar to our patient involving the vertex, parietal, and occipital scalp, but both also had pruritis and dysesthesia [11,12].

The presented patient was asymptomatic and unaware of the scalp swelling, possibly suggesting a chronic process that may have preceded the hair loss. The fact that the scalp thickening and edema spared the frontal scalp begs the question of whether two distinct pathologic processes were present or if this patient had a lipedematous appearance of the non-scarring areas where, clinically, no LPP was noted. High et al. noted a similar presentation of two distinct clinical findings regarding an individual with discoid lupus erythematosus who was found to have LS [7]. Further, LA should be a rule-out diagnosis after diligent evaluation for other causes, as evidenced in a prior case of concomitant LS and alopecia areata [13].

Treatment of LS is primarily symptomatic and utilized in those with dysesthesia, pruritis, and headaches. These symptoms are thought to be caused by mechanical compression of nerves in the scalp [4]. Intralesional steroids have had limited efficacy as a treatment for LA, but topical steroids have been beneficial in clearing alopecic plaques in one case [1,8]. One case with follow-up data reported spontaneous resolution of LS after 13 years [14].

## Conclusions

In the reported case, the presence of significant scalp swelling and thickening may be a reactive process in the context of LPP. This case occurred in a Hispanic/Latino female and highlights the importance of thorough physical examination with palpation of the entire scalp in patients with focal alopecia. Additionally, symptomatic treatment and further workup of scalp edema and hair loss may be necessary in cases such as this, where the alopecia is inflammatory and scarring and there are available therapeutics, unlike in cases of LA. Further studies and case series with long-term follow-up are warranted to establish comorbid associations and treatment recommendations.

## References

[1] Carrasco-Zuber JE, Alvarez-Veliz S, Cataldo-Cerda K, Gonzalez-Bombardiere S (2016). Lipedematous scalp: a case report and review of the current literature. J Dtsch Dermatol Ges.

[2] Müller CS, Niclou M, Vogt T, Pföhler C (2012). Lipedematous diseases of the scalp are not separate entities but part of a spectrum of lipomatous lesions. J Dtsch Dermatol Ges.

[3] Yaşar S, Mansur AT, Göktay F, Sungurlu F, Vardar Aker F, Ozkara S (2007). Lipedematous scalp and lipedematous alopecia: report of three cases in white adults. J Dermatol.

[4] Vasisht S, Bhatia R, Kumar A (2024). Spongy scalp swelling in a middle-aged female: a case report of Lipedematous scalp. Cureus.

[5] Kilinc E, Dogan S, Akinci H, Karaduman A (2018). Lipedematous scalp and alopecia: report of two cases with a brief review of literature. Indian J Dermatol.

[6] Martín JM, Monteagudo C, Montesinos E, Guijarro J, Llombart B, Jordá E (2005). Lipedematous scalp and lipedematous alopecia: a clinical and histologic analysis of 3 cases. J Am Acad Dermatol.

[7] High WA, Hoang MP (2005). Lipedematous alopecia: an unusual sequela of discoid lupus, or other co-conspirators at work?. J Am Acad Dermatol.

[8] Fuentelsaz-del Barrio V, Parra-Blanco V, Borregón-Nofuentes P, Suárez-Fernández R (2012). Lipedematous alopecia in a patient with scalp psoriasis (Article in Spanish). Actas Dermosifiliogr.

[9] Scheufler O, Kania NM, Heinrichs CM, Exner K (2003). Hyperplasia of the subcutaneous adipose tissue is the primary histopathologic abnormality in lipedematous scalp. Am J Dermatopathol.

[10] El Darouti MA, Marzouk SA, Mashaly HM (2007). Lipedema and lipedematous alopecia: report of 10 new cases. Eur J Dermatol.

[11] Fernández-Torres R, Verea Hernando MM, Castro JM, Alvarez R, Fonseca E (2008). Lipedematous scalp: report of a case. J Dermatol.

[12] Martínez-Morán C, Sanz-Muñoz C, Miranda-Sivelo A, Torné I, Miranda-Romero A (2009). Lipedematous scalp. Actas Dermosifiliogr.

[13] Hu D, Yang S (2022). Rare concurrence of alopecia areata in the setting of the lipedematous scalp. Am J Dermatopathol.

[14] Bukhari IA, Bagatadah WA (2016). Spontaneous resolution of lipedematous scalp after 13 years. J Dermatol Plast Surg.

